# Economic connectivity and clustering: The influence of social connections on maternal and infant health

**DOI:** 10.1016/j.ssmph.2025.101794

**Published:** 2025-04-10

**Authors:** Tim A. Bruckner, Samantha Gailey, Brenda Bustos

**Affiliations:** a Joe C. Wen School of Population and Public Health, University of California, Irvine, CA, USA; b Center for Population, Inequality and Policy, University of California, Irvine, CA, USA; c Department of Forestry, Michigan State University, East Lansing, MI, USA; d Department of Public Health, Michigan State University, Flint, MI, USA

**Keywords:** Social networks, Bridging ties, Bonding ties, Facebook, Infant health, Smoking, Zip code

## Abstract

Recent influential work in the US finds that bridging capital (i.e., friendships across socioeconomic strata) appears to increase upward economic mobility over the life course. Examinations of the role of these social connections on health, however, remain relatively unexplored. We exploit a recent effort which made publicly available ZIP-level measures of bridging capital and bonding capital (i.e., depth of friend networks within the same stratum) using Facebook information on >70 million Americans. We test in California (8 million births; California Birth Cohort File 2005 to 2021) the relation between ZIP-level social connections and a wide range of maternal and infant health outcomes, including fetal and infant death, birthweight, preterm birth, pre-pregnancy smoking, and body mass index. Generalized estimating equation methods controlled for individual-level covariates as well as spatial clustering of observations. Findings indicate strong protective associations between bridging capital and the risk of fetal death, infant death, preterm birth, pre-conception maternal smoking, maternal body mass index, and infant birthweight. These protective associations are much greater in magnitude than other ZIP-level measures commonly used in the literature to assess economic and structural advantage. Bonding capital, however, appears detrimental for maternal pre-pregnancy smoking and body mass index. The potential of bridging ties (i.e., across socioeconomic strata) in promoting maternal and infant health should warrant much more scholarly consideration than it currently receives.

## Highlights

• Having friends across socioeconomic groups may benefit maternal and infant health.
• Bonding social ties, by contrast, show some health-damaging associations.
• Future work should examine whether bridging social ties reduce health disparities.

## 1 Introduction

Of all high-income countries which routinely report data, the infant mortality rate and the risk of maternal death related to complications of pregnancy and childbirth in the US ranks among the worst (Tikkanen et al., 2020; United Health Foundation, 2023). Aside from mortality, the risk of preterm (i.e., delivery before 37 weeks of completed gestational age; PTB) and growth restriction (often measured as low birthweight [<2500 g]; LBWT) also remains elevated in the US relative to other high-income countries (United Health Foundation, 2024). Infants born preterm and/or LBWT, moreover, show an increased risk of infant death, asthma, impaired cognitive development, and hyperactivity (Black et al., 2007, Boardman et al., 2002, Hack, Klein, & Taylor, 1995). These conditions also predict reduced human capital in adulthood, including lower educational attainment and adult earnings (Black et al., 2007; Hack et al., 1995). For these reasons, improving maternal and infant health in the US remains a key federal priority.

Much literature in the social sciences and public health contends that the social environment may promote the formation of human capital—including health (Putnam, 2000; Villalonga-Olives & Kawachi, 2017). Aspects of the social environment include not only social structures (e.g., political systems of redistribution, opportunities for exchange and interaction) but also social cohesion (Lomas, 1998). Social cohesion refers to connectedness and solidarity among groups (Kawachi & Berkman, 2000).

The lack of large-scale data (Jeon & Goodson, 2015) has severely limited our understanding of the role of social connections on maternal and infant health. The recent availability, however, of such data in the U.S. (Chetty et al., 2022a, Chetty et al., 2022b) makes it possible to examine whether, and to what extent, group-level measures of social connections relate to maternal and infant health. In this paper, we assess this relation using the universe of California birth records over a 16-year period.

## 2 Background

### 2.1 Social connections and maternal and infant health

Population health researchers generally define social capital as either a collective resource—often operationalized as “social cohesion”—or an individual attribute—referred to as the “social network” approach (Kawachi & Berkman, 2000). Like Chetty et al. (2022a), our study views social capital as an important resource that community members acquire through involvement with their social networks (Bourdieu, 1986). From this perspective, social capital may promote health via social networks by providing members with opportunities to learn new skills and transfer knowledge, gain access to social support and material resources, and through the social influence of role models and exposure to health-reinforcing norms (Berkman et al., 2000; Eriksson & Ng, 2015). A meta-analysis of social capital and health suggests a strong positive association between area-level social networks (e.g., measured at the neighborhood, state, and national level) and self-rated health (Gilbert et al., 2013). Chetty et al. (2022a) and others (e.g., Jackson, 2020) have further distinguished several types of network-based measures of social capital, including bridging and bonding capital, but their unique impacts on health appear equivocal (Gilbert et al., 2013; Iwase et al., 2012).

Whereas causes of LBWT and PTB (among other perinatal outcomes) remain poorly understood, cross-sectional evidence indicates that social supports may reduce their risk (Amjad et al., 2019). For instance, US-based work using a population-based sample (i.e., National Survey of Family Growth) finds that partner involvement early in the pregnancy may reduce risk of PTB (Shah et al., 2014). In addition, a smaller study of Mexican-born US women reports a positive association between social supports and salutary health behaviors during pregnancy (e.g., better diet, reduced smoking (Harley & Eskenazi, 2006). Other evidence from outside the US, reviewed by Mengesha and colleagues (2021), uses small samples (i.e., <1000 participants). This work generally finds that “bridging ties”—that is, social connections to out-group members— may improve access to maternal and child health services. Qualitative findings further indicate that pregnant persons may have, through their bridging ties, received some form of emotional, informational, and instrumental support from their network members (Mengesha et al., 2021).

The literature regarding the role of bonding ties—that is, strong relations among people with similar characteristics—on maternal health during pregnancy relies on small sample sizes and shows mixed results (Kritsotakis, 2011; Tofani et al., 2015). Explanations for reported salutary, and some detrimental, associations include the fact that in-group norms regarding health behaviors (e.g., smoking) may differ across social contexts. Depending on these norms, having strong social connections could operate to either increase or reduce likelihood of that behavior.

In summary, the literature reports associations between social supports and maternal and child health. Extant work in this area, however, uses relatively small samples and does not distinguish between bridging social connections and bonding ties. Literature examining other life stages demonstrates the importance of peer networks on health behaviors (e.g., smoking and unhealthy eating; see (Christakis & Fowler, 2007; Conrad, Flay, and Hill, 1992; Flay et al., 1998; O'Loughlin et al., 1998). Despite the potential importance of the role of bridging and bonding connections on maternal and infant health, the lack of large-scale data on social connections has heretofore precluded such an examination.

### 2.2 Social connection data

Chetty and colleagues (2022a) recently conducted a large-scale project on social networks in the US. By analyzing data on 72.2 million users of Facebook aged 25–44 years, they generated social connection measures for individuals as well as for each aggregated ZIP code tabulation area (ZCTA) in the US. Two key social connection measures that they derived include economic connectivity (EC) and clustering.

EC assesses bonding ties across levels of socioeconomic status (SES), or the degree to which people with low and high SES are friends. Chetty and colleagues initially measured EC and clustering at the individual level but then operationalize them at the small-scale ecological level of a ZCTA (i.e., ZIP code tabulation area). ZCTAs represent geographic areas that closely approximate ZIP codes. The US Census Bureau created ZCTAs to enable standardized data analysis, since the US Postal Service developed ZIP codes primarily for mail delivery purposes that do not necessarily align with research needs (US Census Bureau, 2023). Chetty and colleagues(2022a) and others use the terms ZCTA and ZIP code interchangeably; both units of analysis receive popular use in the literature as area-level measures of social capital (Gilbert et al., 2013).

ZCTA-level EC is defined as “the average share of above-median-SES friends among below-median-SES members of that community divided by 50 %… [a] value of 0 for EC implies that a network has no connections between low-SES and high-SES people, whereas a value of 1 implies that low-SES people have an equal number of low-SES and high-SES friends” (Chetty et al., 2022a). Separately, clustering aims to capture a form of bonding capital, which refers to the depth of friend networks. Chetty and colleagues operationalize clustering as the rate at which two friends of the same person are friends with each other. The concept of clustering, as a form of bonding capital, appears quite distinct from EC (a form of bridging capital) because deep bonds may serve to reinforce social norms, intensify peer pressure, and/or promote time investment in that group. We note, however, that EC and clustering need not be inversely correlated by axiom at the ZCTA level because high clustering of friends could--in principle--occur across low- and high- SES strata.

The authors assumed that Facebook connections proxied real-world friendships. To cross-validate this assumption, Chetty and colleagues compared their measures of EC and clustering, from Facebook data, with measures of EC and clustering derived from Add Health data. They use Add Health data given that it represents the most commonly used dataset in the US to research social networks, includes a range of geographic contexts, and shows a range of SES backgrounds among friends (Harris et al., 2019, Harris and Richard Udry, 2018). The agreement in friend sorting by SES, and in friend preference by SES, between Facebook- and Add Health-derived results is quite high, which led Chetty and colleagues to conclude that any potential selection bias in Facebook usage (relative to “real-world” friendships from Add Health) cannot substantially distort their estimates. Put another way, they observe no meaningful difference in the social network characteristics (across SES) between Facebook and Add Health data. The fact that the Facebook data capture 3500 fold more persons than does Add Health allows for the creation of ZCTA-level measures of social connection not previously possible with smaller datasets.

Chetty and colleagues focused their empirical analyses on whether certain aspects of social connections predicted upward economic mobility from childhood to adulthood. They find that economic connectedness (EC)—that is, the degree to which people with low and high socioeconomic status (SES) are friends—strongly predicted upward economic mobility (i.e., rising SES from childhood to adulthood). The authors reasoned that connections of low SES persons to more resourced persons could assist with transferring information, providing job and economic opportunities, and shaping aspirations. By contrast, the authors find that clustering—a measure of social connection which is measured by the rate at which two friends of a given person are in turn friends with each other—showed no relation to upward economic mobility.

### 2.3 Hypotheses

The literature cited above supports the plausibility that area-level 10.13039/501100000780EC and/or clustering measures of social connection could predict improved maternal and infant health. We, as with Chetty and colleagues, assume that ZCTA-level EC and/or clustering (as measured by Facebook) captures real-world ZCTA-level social interactions. ZCTA-level EC could benefit maternal and infant health by promoting access to appropriate health care or information before and/or during pregnancy, which in turn could improve the course of pregnancy. Alternatively, ZCTA-level EC could promote maternal and infant health indirectly through the upward economic mobility channels discovered by Chetty and colleagues (e.g., improved SES via greater employment attainment). Another plausible pathway by which EC could benefit health would involve interacting with peers who exhibit relatively healthier behaviors (e.g., less smoking among high-SES persons).

The potential pathways connecting ZCTA-level clustering and maternal and infant health could take several forms. 10.13039/100014345Strong bonding ties, for instance, could signal relatively high social support before and during pregnancy, which in turn could buffer against psychosocial stressors and improve maternal and infant health. By contrast, if bonding ties connect persons to networks with unhealthy behaviors, such ties could adversely affect maternal and infant health.

Based on the above logic, we propose two hypotheses.

1. ZCTA-level EC will vary positively with a broad set of maternal and infant health indicators, as well as maternal health behaviors.
2. ZCTA-level clustering will vary positively with a broad set of maternal and infant health indicators.

The specific indicators we study include fetal death, infant death, preterm birth, birthweight, maternal pre-pregnancy smoking, and maternal body mass index. Given that that ZCTA-level EC and clustering may show either a positive or inverse association with these indicators, we specify all tests as two-tailed. We test these predictions using the universe of maternal and infant health data from California—the most populous state in the US—over a 17-year period (2005–2021). Given the wide range of mechanisms by which ZCTA EC and clustering could affect maternal and infant health, we analyze a broad set of maternal and infant health outcomes available in the California Birth Cohort files. We, moreover, control for individual level sociodemographic factors known to affect maternal and infant health (Iams et al., 2008; MacDorman, 2011). Lastly, we compare the predictive ability of social connection measures on maternal/infant health relative to numerous other area-level measures used in the literature that capture social and economic deprivation and aspects of structural inequality.

Our descriptive analysis contributes to the literature in three ways. First, it represents the first rigorous application (to our knowledge) of the novel ZCTA social connection dataset to maternal and infant health. Second, we improve upon the existing social ties and infant health literature by using a population base that is at least 500-fold larger than previous studies (Hetherington et al., 2015; Nkansah-Amankra et al., 2010) and by controlling for a wide range of individual-level variables when examining the area-level influence of social connection. Third, by offering direct comparisons of the predictive power of social connection measures relative to “conventional” area-level measures of social and economic conditions (e.g., Gini coefficient, percent poverty, median household income), our work may inform whether area-level social connection measures deserve more attention from neighborhood scholars than what they currently receive (Morenoff, 2003).

## 3 Methods

### 3.1 Variables and data

We retrieved live birth and infant death data from the California Department of Public Health (CDPH) Birth Cohort Files for the years 2005–2021. The Birth Cohort Files compile birth certificates and infant death reports. Each file contains all live births that occurred during the calendar year, including all state registered births and births to California residents that occurred out-of-state, linked (when applicable) to death information for infants that subsequently died within 12 months of birth.

We also retrieved data on fetal deaths for the years 2005–2021 from the Birth Cohort File with the exception of the years 2013–2015 in which we obtained fetal deaths from the Fetal Death Statistical Master File. The State of California requires reporting of all fetal deaths (“stillbirths”) after 20 weeks of gestational age, except for in the case of induced abortions. A fetal death is indicated, “… by the fact that the fetus does not breathe or show any other evidence of life such as beating of the heart, pulsation of the umbilical cord, or definite movement of voluntary muscles” (California Department of Health ServicesOffice of Vital Records, 1996; Gaudino et al., 1997; Gould, 1999; Martin & Hoyert, 2002).

The 17-year study period uses consistent definitions and reporting for live births, infant deaths, and fetal deaths, and contains nearly 100 % of all birth/death events (California Department of Health Services Center for Health Statistics, 2004, California Department of Health ServicesOffice of Vital Records, 1996; Catalano et al., 2005). For the years 2005–2021, the Birth Cohort Files recorded information on N = 8,502,718 live births (of which N = 40,457 converted into infant deaths) and recorded N = 44,116 fetal deaths. The total population of live births and fetal deaths in California, 2005–2021, includes N = 8,546,834 events. The institutional review boards at the California Department of Public Health (# 2018-065) and the University of California, Irvine (# 2013-9716) approved the use of these data for our study.

The Birth Cohort Files include demographic information related to the infant/fetus and parents, medical data related to the pregnancy and birth, and geographic identifiers for the mother's place of residence. Since our key independent variables—social connection exposures (EC and clustering)—in California remain at the ZCTA level, we excluded live birth/fetal death records missing information on maternal ZCTA of residence (N = 37,706) and residing in ZCTAs outside of California (N = 24,400).

We retrieved ZCTA EC and clustering, for California only, directly from the publicly available source (socialcapital.org). We refer interested readers to Chetty and colleagues (2022a) for a detailed description of the construction of EC and clustering from 72 million Facebook users, measurement validity checks, and other data robustness checks. We merged the Birth Cohort File data to the EC and clustering data by ZCTA.

### 3.2 Measures

#### 3.2.1 Area-level characteristics

Prior work using Facebook data examines the relation between social networks and a variety of outcomes, including health (e.g., (Allen, Lin, & Shan, 2024; Bailey et al., 2020; Hobbs et al., 2016; Kuchler et al., 2022). The primary analysis sample Chetty and colleagues use to construct EC and clustering measures consists of Facebook users residing in the US and aged between 25 and 44 years. The users must have remained active on the Facebook platform at least once in the prior 30 days, report at least 100 U.S.-based Facebook friends, and have a non-missing residential ZIP code. In addition, Facebook data show population-representativeness, in terms of geographic and sociodemographic coverage, that matches figures from the US Census and American Community Surveys characteristics (see Extended Data Table 4 within the supplemental information of Chetty and colleagues’ (2022a)article). This circumstance lends measurement validity to the ability to use Facebook-derived measures to compare social connections across place.

Economic connectedness (EC) refers to the extent to which individuals are friends with persons above and below their SES. The key assumption, which is validated using Add Health data (Chetty et al., 2022a, Chetty et al., 2022b), involves the notion that Facebook friend connections approximate real-world friendships. ZCTA level EC is defined as two times the share of high-SES friends among low-SES individuals, averaged over all low-SES individuals in the ZCTA. A greater EC value in that ZCTA indicates a relatively greater share of high-SES friends among low-SES adults (relative to other ZCTAs). EC serves as a form of “bridging capital” in that it reflects outward looking connections that include persons from diverse social strata (Putnam, 2000).

Clustering—a measure of “bonding capital” that reflects inward-looking networks within a social stratum (Putnam, 2000)—is also derived from the Facebook dataset. Chetty and colleagues define clustering as the average fraction of an individual's friend pairs who are also friends with each other. The ZCTA measure of clustering is the average of individual-level clustering values over users in the relevant ZCTA. Clustering may permit strong social supports but also holds the potential to reinforce norms considered harmful to society (Patulny and Svendsen, 2007).

Several other area-level measures capture neighborhood social and economic structure and appear in the literature on maternal and infant health (Gailey et al., 2024). Existing social and economic structures not caused by EC or clustering may exert an important influence on maternal and infant health. Given that the relation, if any, between EC or clustering with maternal and infant health remains unknown, we plotted the predictive ability of EC and clustering along with the predictive ability of these other measures. We therefore collected ZCTA information on the following social and economic measures frequently used in the literature (and, to permit comparisons to prior work (Chetty et al., 2022a, Chetty et al., 2022b): Gini Coefficient, Median Household Income, Percent Below Poverty, Proportion Black (all from the 2007–2011 American Community Survey (ACS), and Proportion Single Parent (from the 2010 US Census). Using only the ZCTA information in California (n = 1763 ZCTAs), we then converted each area-level metric to a standardized Z-score (i.e., number of standard deviations above or below a mean value, in which mean = 0 and standard deviation = 1). This standardization permits direct comparison of area-level coefficients on maternal and infant health across separate regression specifications.

#### 3.2.2 Maternal and infant outcomes

Six variables, retrieved from the Birth Cohort Files—including four binary and two continuous variables—served as primary outcomes in logistic and linear regression models, respectively. Binary outcomes included infant death, fetal death, PTB, and pre-pregnancy smoking. As described above, infant death refers to the death of an infant (born live) within the first year of life, whereas fetal death refers to a “stillbirth,” or a “death prior to the complete expulsion of extraction from its mother of a product of human conception […] indicated by the fact that the fetus does not […] show any evidence of life” (California Department of Health Services Office of Vital Records, 1996). PTB includes live-born infants delivered at gestational ages of less than 37 completed weeks, which we derived using the clinical estimate of gestation available on the birth record.

In 2006, CDPH began collecting data on maternal smoking behavior in four separate epochs: three months prior to pregnancy, and the first, second, and third trimester of pregnancy. Here, we use smoking in the three months prior to pregnancy (indicated by a response that includes “at least one cigarette per day”). We used pre-pregnancy smoking since, of all smoking variables available in the data, pre-pregnancy smoking shows the greatest coverage across data years and the lowest frequency of missingness. We categorize maternal pre-pregnancy smoking for years 2006–2021 as a categorical (yes/no) variable.

Continuous outcomes specified in linear regression analyses included maternal pre-pregnancy body mass index (BMI) and infant birthweight. In 2007, CDPH adopted the Revised U.S. Standard Certificate of Birth, which instituted the collection of maternal weight and height data. We calculated maternal pre-pregnancy BMI, for years 2007–2021, as pre-pregnancy weight (in kilograms) divided by height (in meters) squared. Lastly, the U.S. Standard Certificate of Birth (Revised and Unrevised) records infant birthweight in grams at the time of birth. For clarity of presentation of results, we classify a gain in BMI as a less desirable (i.e., “harmful”) outcome given the high prevalence of overweight/obesity in California. That stated, we cannot with certainty identify in the aggregate results whether a gain in BMI associated with, say, clustering, would harm health. As for infant birthweight, given that lower birthweights typically correspond with worse health, we classify a decline in birthweight as a less desirable (i.e., “harmful”) outcome when characterizing its association with EC and clustering. Conversely, we refer to reductions in BMI and gains in infant birthweight as “protective” for health throughout the remainder of the manuscript.

#### 3.2.3 Sociodemographic covariates

The Birth Cohort Files include several individual demographic and socioeconomic characteristics of the infant and mother, which we used as covariates in analyses, including: infant sex (male, female, or other/not stated), maternal age (continuous age and continuous age-squared), highest educational attainment (categorized as less than high school diploma, high school diploma, some college or more, or not stated), expected insurance payer (categorized as private, public/Medi-CAL [California's version of Medicaid], other [including self-pay, Indian Health Service, other governmental source, and unknown or not stated]), receipt of the Specialized Supplemental Nutrition Program for Women, Infants, and Children (WIC; categorized as yes, no, or not stated), and maternal race/ethnicity, which we defined, consistent with the literature (Gailey et al., 2021, 2024), as non-Hispanic (NH) White, NH Black, Hispanic, Asian, and other.

### 3.3 Statistical analysis

We conducted a cross-sectional study. We first examined descriptive statistics for the population of live births and fetal deaths in California, 2005–2021, including sociodemographic and ZCTA characteristics. Then, we specified logistic regression analyses (for binary outcomes) and linear regression analyses (for continuous outcomes) to estimate (separate) associations of seven ZCTA-level independent variables—including two key social connection exposures: EC and clustering; and five comparison exposures: Gini Coefficient, Median Household Income, Percent Below Poverty, Proportion Black, and Proportion Single Parent—with maternal and infant health outcomes (infant death, fetal death, PTB, pre-pregnancy smoking, pre-pregnancy BMI, and infant birthweight).

Exclusion criteria differed across outcomes.

(i.) All analyses excluded mothers aged less than 15 years or over 50 years and non-singleton births;
(ii.) fetal death analyses additionally excluded infant deaths;
(iii.) infant death, PTB, and pre-pregnancy smoking analyses additionally excluded fetal deaths;
(iv.) pre-pregnancy BMI analyses additionally excluded fetal deaths and live births to mothers with BMI less than 15 or greater than 50; and
(v.) infant birthweight analyses additionally excluded fetal deaths and live births in which the infant weighed less than 400 g or greater than 4500 g.

All analyses controlled for infant sex, maternal age, highest educational attainment, expected insurance payer, and maternal race/ethnicity. We restricted the analysis to singletons. In addition, owing to lack of data availability, we could not control for marital status. Given Chetty and colleagues’ results of the relative homogeneity across race/ethnicity of results regarding the role of EC and clustering on upward economic mobility, we decided to include individual race/ethnicity as a covariate rather than as a variable on which to stratify analyses. We plotted results of all 42 regression models (7 ZCTA-level exposures x 6 outcomes, with each ZCTA-level exposure run separately) in forest plots.

We conducted all analyses using SAS version 9.4 (Cary, North Carolina). The GENMOD procedure fit logistic regression analyses for binary outcomes (using the link “logit” function and binary response probability distribution) and linear regression analyses for continuous outcomes (using the normal response probability distribution). Given the potentially correlated nature of individual observations within the higher ZCTA level, we assumed an exchangeable working correlational structure (recommended in the literature (Hubbard et al., 2010) and specified robust standard errors in the maximum likelihood estimations using the “REPEATED” option in SAS). In circumstances where maximum likelihood estimation did not converge (owing to the relatively low within-ZCTA correlation of outcomes and the large sample size of the CA birth file), we specified standard error estimation assuming independence (i.e., no clustering of individual observations within ZCTA).

## 4 Results

Table 1 describes the sociodemographic characteristics of the 8 million Birth Cohort File records in California from 2005 to 2021. Over 49 % of all recorded pregnancies occurred among Hispanic birthing persons. Private health insurance appears the most reported insurance coverage for pregnancy and delivery. The prevalence of preterm (i.e., <37 weeks gestational age) is 8.7 per 100 live births. The infant death rate is 4 per 1000 live births, and the fetal death rate is 5 per 1000 recorded pregnancies (sum of fetal deaths and live births).

**Table 1:** Maternal and birth characteristics among birthing person ages 15 to 50 with singleton births in California Department of Health Services Office of Vital Records, 1996 to 2021.

	*Births* (*N = 8,265,759*)	
	N	%
Preterm Birth (<37 weeks)	699,263	8.7
Fetal Death	39,562	0.5
Infant Death	34,013	0.4
Smoking Before Pregnancy	192,628	2.7
Maternal BMI (mean, SD)	26.3 (6.1)	–
Birthweight (mean, SD)	3316.7 (562)	–
Infant Sex		
Male	4,235,466	51.2
Number of previous live births		
0	3,289,693	39.8
1-2	4,011,857	48.6
3+	955,667	11.6
Race/ethnicity		
Non-Hispanic White	2,293,820	27.8
Non-Hispanic Black	452,876	5.5
Hispanic	4,082,377	49.4
Non-Hispanic Asian	1,146,581	13.9
Other	290,105	3.5
Maternal Age (yrs)		
<18	165,701	2.0
18-25	2,390,681	28.9
26-34	4,062,816	49.2
35+	1,646,561	19.9
Maternal Education		
<High School	1,636,611	20.7
High School Graduate	2,028,991	25.7
>High School	4,245,490	53.7
Health Insurance		
Medi-Cal	3,701,441	44.8
Private	3,916,953	47.4
Other	638,128	7.7
Enrolled in WIC	3,429,721	48.3

Table 2 shows the mean, standard deviation, and range of Z-scored ZCTA measures, which serve as the key independent variables. The Z-standardization produces means of 0 and standard deviations of 1. The ACS-derived measures, as well as the Facebook-derived social connection measures, show substantial range (and often skew—see maximum for proportion Black, for example) across the 1763 California ZCTAs.

**Table 2:** Descriptives of Z-scored measures of social capital and neighborhood characteristics across 1763 California zip-codes.

	Mean (SD)	Min	Max
Economic Connectivity	−0.006 (0.998)	−2.111	3.146
Clustering	0.0001 (1.001)	−2.995	16.837
Gini Coefficient	−0.002 (0.998)	−9.577	7.280
Median Household Income	−0.006 (0.996)	−2.587	7.882
Percent Below Federal Poverty Level	0.005 (1.000)	−1.787	9.573
Proportion Black	0.0004 (1.000)	−0.760	9.521
Proportion Single Parent	0.004 (1.000)	−3.401	7.795

**Fig. 1:**
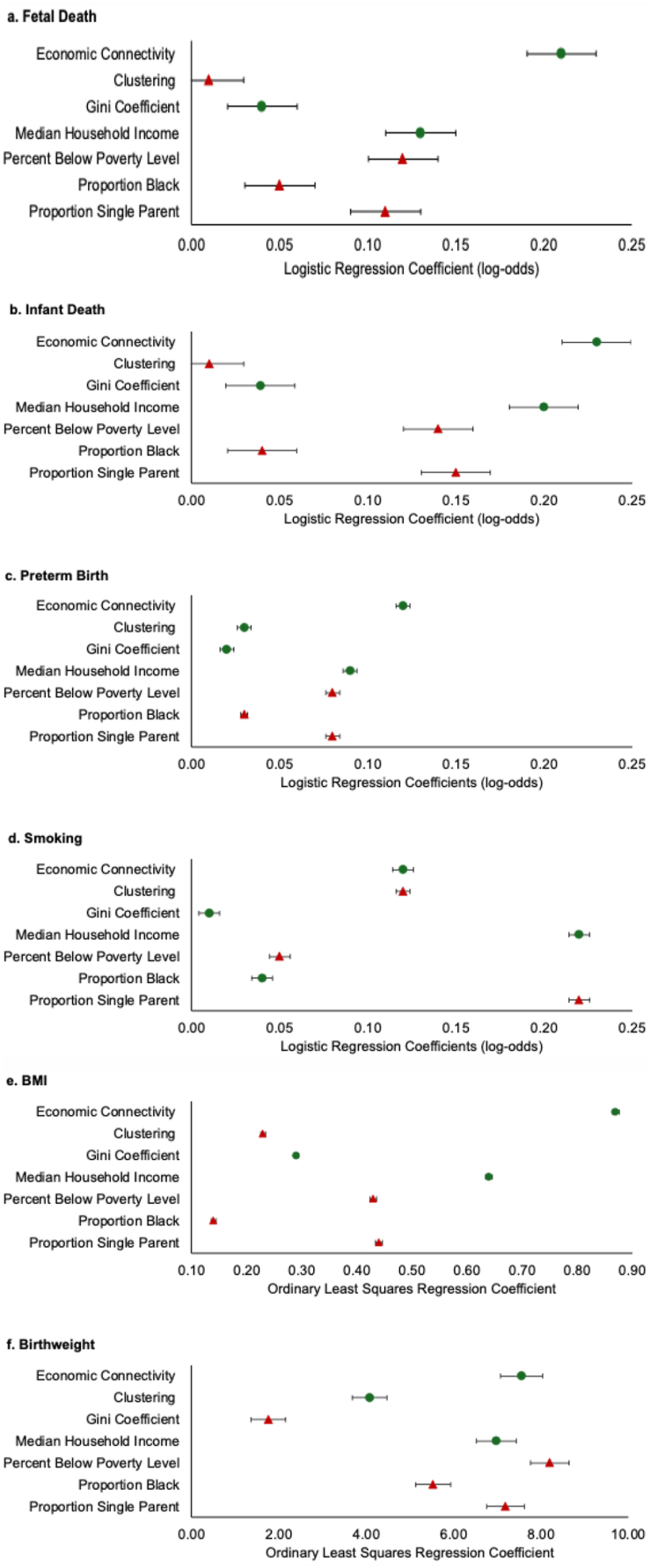
Zip code level measures of social capital and neighborhood characteristics and their association with maternal/infant health outcomes among births in California. **Green circles** represent protective associations. **Red triangles** represent harmful associations. The 95 % Confidence Intervals of coefficients are shown with whisker plots (all but d. Smoking Confidence Intervals are estimated with robust standard errors that control for correlated observations at the ZCTA level).

Fig. 1 (Panels A through F) show our main results; we report all analytic samples in Appendix A. In all models save for pre-pregnancy smoking (Panel D), the robust standard error maximum likelihood estimation strategy converged. For pre-pregnancy smoking, however, lack of convergence forced us to use OLS assumptions to derive standard errors. Each panel displays coefficients estimating a distinct maternal or birth outcome; the coefficients of all individual covariates are included in all regressions but are not shown in the plot (see Appendices B through H for full set of regression coefficients for the outcome of fetal death). Protective associations appear in green circles, and harmful associations appear in red triangles. Within each panel, each row highlights the ZCTA-level coefficient (and 95 % CI) for that panel's health outcome. Put another way, each panel reflects the output from seven separate regressions (i.e., seven separate rows) in which we include (one at a time) the ZCTA measure of interest.

For each of the six outcomes examined, EC shows a statistically detectable protective association. The protective associations of EC are larger in magnitude than any other ZCTA measure for five of the six health outcomes (i.e., fetal death, infant death, PTB, maternal BMI, and infant birthweight). For the one outcome (maternal smoking) in which EC does not show the most protective association, median household income appears most protective.

Results between clustering and maternal/infant health show much more nuance than do the EC findings. Clustering shows no association with fetal and infant death but protective associations with PTB and birthweight. Interestingly, greater ZCTA clustering corresponds with (harmful) greater maternal BMI and smoking. The BMI and smoking results contrast the protective associations observed with EC.

All other ZCTA measures of social and economic structure and inequality show associations with maternal/infant health that cohere with predictions from prior literature (Chambers et al., 2019; Das et al., 2024; Huynh et al., 2005; Nkansah-Amankra et al., 2010; Wallace et al., 2016). Median household income varies positively with better health outcomes, especially relative to the other non-social connection measures. Although the remainder of the ZCTA measures do not show consistent patterns across all six maternal/infant health outcomes, the proportion single parent also tends to show harmful associations of moderate magnitude.

We conducted two additional analyses to assess of the main EC and clustering results to alternative specifications. First, we excluded observations with high or low ZCTA outliers in EC and/or clustering (i.e., dropped observations with Z-scores greater than absolute value of 3.0). Here, we examined all six health outcomes and two regressions per outcome—EC and clustering (i.e., 12 checks total). Inference remains essentially similar to the original results (see Appendix I). Second, for the two continuous outcomes which could have outlier values (i.e., BMI and infant BWT), we re-ran the EC and clustering analyses but retained ALL values of BMI and BWT (i.e., we did not exclude implausible values). Results do not differ appreciably from the original regression results reported in Fig. 1 (see Appendix I). We also include descriptives of ZCTA-level social connections and other socioeconomic characteristics (see Appendix J).

## 5 Discussion

The recent availability of measures of social connections in the US, using large population samples, provides a unique opportunity to examine the extent to which area-level social connections could affect maternal and infant health. We contribute to the literature by examining in California whether economic connectedness (EC) and clustering—measures of bridging and bonding ties, respectively—vary positively with maternal and infant health. Results using over 8 million birth records and social connection data from 1763 ZCTAs show strong protective associations of EC with infant and maternal health. By contrast, clustering does not appear consistently protective and sometimes even shows harmful associations. Taken together, our findings should encourage much more attention than currently given by sociologists and population health scholars to the potentially health-promoting role of economic connectedness (Linde & Egede, 2023).

Previous work speculates on potential mechanisms connecting social networks to maternal and infant health outcomes. Shah et al. (2014) suggest that strong social support may improve perinatal outcomes by reducing maternal stress and encouraging early initiation of prenatal care. In addition, Harley and Eskenazi (2006) highlight the role that social networks play in the uptake and continuation of healthy pregnancy behaviors (e.g., use of prenatal vitamins, smoking cessation). This work, however, appears limited in that it does not attempt to differentiate between distinct characteristics of social connections, including bonding and bridging capital, and how each operates uniquely on perinatal health. Whereas we do not empirically examine specific mechanisms linking bridging and bonding capital to perinatal health (and thus caution readers that the hypotheses below require further refinement and testing), findings indicating divergent associations of economic connectedness and clustering with perinatal outcomes may support that these forms of social capital operate through distinct pathways to improve or, in some cases, degrade maternal and infant health.

We find, for instance, that economic connectedness (EC), or the degree to which low and high SES people are friends, shows protective associations with all six maternal and infant outcomes of interest. By contrast, clustering, or strong ties among people of similar SES, shows null relations with fetal and infant death and harmful associations with BMI and smoking. Prior work leads us to speculate that the promotion of health-reinforcing norms and transmission of knowledge and shared resources across high-to low-SES people represents a core set of pathways through which EC positively affects maternal and infant health (Mengesha et al., 2021). For example, pregnant people of high SES appear more likely to initiate prenatal care earlier, which in turn varies with a range of salutary maternal and infant health outcomes (Gadson et al., 2017; Green, 2018). Exposure to early prenatal care or knowledge of its benefits as disseminated through “bridged” social networks may increase the likelihood that pregnant people of low SES engage in this and other health-reinforcing behaviors. Similarly, friendship with pregnant people of high SES may serve as a critical point of contact through which those of low SES learn about and connect with key health services and resources, including not only prenatal providers, but also postpartum care, immunizations, and parenting classes that reduce risks of adverse infant and childhood outcomes (Story, 2014).

By contrast, clustering may reinforce deleterious social norms that may permeate low-income networks and govern adverse health behaviors, such as poor diet and pre-pregnancy smoking. Evidence of the contagion of obesity (Christakis & Fowler, 2007) and smoking initiation and continuation (Kobus, 2003) through peer networks appears to support this hypothesis, albeit in non-pregnant populations. Bonding capital may also restrict individual autonomy to make positive health care decisions that break from social norms (Story, 2014). Further research on the mechanisms linking EC and clustering to maternal and infant outcomes appears warranted to understand whether and how these unique aspects of the social environment may contribute to health above and beyond more “classical” indicators favored by the literature.

We note, moreover, that the mechanisms that hold relevance for maternal and infant health may differ from those that influence economic mobility. Chetty et al. (2022a), for example, suggest that bridging capital may promote upward mobility by providing job opportunities and shaping career aspirations—factors that may act “upstream” of perinatal health (Gailey et al., 2022, Gailey et al., 2022). Other mechanisms, including the transfer of knowledge, may support a range of positive outcomes, though the type of information exchanged (e.g., medical vs. employment) may differ.

EC within a community depends on both the prevalence of high SES persons and the probability of mixing among lower- and higher- SES persons (Chetty et al., 2022b). For this reason, it seems logical that median household income—another ZCTA measure—shows protective associations on maternal/infant health that are similar (albeit generally smaller in magnitude) to that of EC. An intuitive follow-up question to our work would involve assessing the extent to which EC varies positively with maternal and infant health even in settings in which median household income for that ZCTA is relatively low. Given the strong stratification of household income by place (Osypuk & Acevedo-Garcia, 2010; Osypuk et al., 2009), however, we await future empirical investigations to determine the feasibility of such analyses that focus on EC in areas characterized by relatively few high-SES persons.

Strengths of the study include examining a broad set of important maternal/infant health outcomes for the population base of records in the California Birth Cohort file over a 17-year period. This circumstance, combined with the broad ZCTA coverage of EC and clustering measures, permits population-level inference and increases precision of results. Direct comparison of social connection measures to standardized area-level indicators of social and economic structure, moreover, permits an evaluation of the extent to which novel social connection measures outperform (in terms of predictive ability) “classical” indicators used in the literature (Hetherington et al., 2015; Nkansah-Amankra et al., 2010). In addition, the use of Chetty and colleagues’ social connection data resources, with a very large sample and broad ZCTA coverage, vastly improves upon earlier studies that assess area-level social connections.

The ability to estimate associations among distinct constructs of bridging ties and bonding ties, relative to prior work that often uses the broader term of social supports, further improves upon the literature. The fact that we discovered opposite, and often countervailing, associations for EC and clustering underscores the possibility that these types of social connections affect health via distinct pathways. Nevertheless, given that we cannot link individual-level data on EC and clustering to BCF records, we hesitate to draw any inference about mechanistic pathways. We, instead, view the salutary associations between area-level EC and a broad array of individual level maternal and infant health outcomes as evidence to further scrutinize, using other datasets, the extent to which bridging ties with higher-SES groups could promote health. The fact, moreover, that area-level EC routinely shows stronger protective associations than do “classical” area-level measures of social and economic inequality should encourage scholars to study the extent to which societal and organizational structures may promote, or hinder, EC (Chetty et al., 2022b).

Limitations involve the correlational nature of our cross-sectional inquiry and the inability to establish temporal order between exposure and outcomes, which precludes causal inference of any role of EC on maternal and infant health. We cannot know, for instance, whether persons at lower risk of fetal death selectively move to neighborhoods with high EC (or clustering). This endogeneity rival, also referred to as reverse causality, remains a viable threat to inference given well-documented health selection processes across neighborhoods (Gailey et al., 2021; Oakes, 2004; Osypuk et al., 2024; Rolheiser et al., 2022). Such selection, however, would have to occur independently of sociodemographic individual level variables (e.g., Medi-Caid health insurance, maternal education, race/ethnicity, maternal age) because we controlled for them in our models.

We also do not know how long the birthing person resides in the current ZCTA owing to lack of data on full residential histories. Future data collection efforts may want to consider how residential moves, and duration of exposure to high or low EC regions, affect maternal/infant health. We also caution against direct comparison of coefficients across the outcomes studied given that we retained some in continuous form and others in categorical form. In addition, whereas our robust standard error estimation methods control for clustering of observations within a ZCTA, we do not adjust estimates for spatial autocorrelation *across* contiguous ZCTAs. This circumstance makes a simplifying assumption that adjacent ZCTAs do not affect the relation of within-ZCTA exposures and health outcomes. Given that EC and clustering may show strong spatial autocorrelation across ZCTAs, we encourage future work to assess and control for this possibility.

In a separate analysis of cardiovascular health among adults in the US, Linde and Egede (2023) report protective associations of EC yet mixed results for measures of bonding capital. Although we caution against comparing our findings directly to theirs (owing to different populations and outcomes studied), their pattern appears broadly consistent with ours studying maternal and infant health in California.

We hesitate to speculate on the implications of our work, given the study design caveats noted above. Nevertheless, on the supposition that results may in part reflect a benefit of EC on health, previous literature has argued that multiple mechanisms could play a role (Amjad et al., 2019; Harley & Eskenazi, 2006; Shah et al., 2014). Transmission of health information and promotion of health-reinforcing norms across high- and low-SES persons could improve health especially among lower-SES persons. Alternatively, the ability to connect to greater employment and educational opportunities may improve household income among previously low-SES persons. We note, however, that these potential pathways remain purely speculative and merit much more refinement and testing. In addition, we encourage future investigations regarding the extent to which EC and clustering correspond with maternal and infant health after jointly considering the role of other ZCTA characteristics (e.g., median household income).

## Funding

This research was supported in part by Eunice Kennedy Shriver National Institute of Child Health and Human Development (R01 HD103736, PI TAB).

## CRediT authorship contribution statement

**Tim A. Bruckner:** Writing – review & editing, Writing – original draft, Validation, Investigation, Formal analysis, Conceptualization. **Samantha Gailey:** Writing – review & editing, Writing – original draft, Investigation, Formal analysis. **Brenda Bustos:** Writing – review & editing, Writing – original draft, Visualization, Investigation.

## Ethical statement

The institutional review boards at the California Department of Public Health (# 2018–065) and the University of California, Irvine (# 2013–9716) approved the use of these data for our study.

## Declaration of competing interest

The authors declare no conflicts of interest.

## Figures and Tables

**Table:** 

	Economic Connectivity (N)	Clustering (N)	GINI Coefficient (N)	Median Household Income (N)	Proportion Poor (N)	Proportion NH Black (N)	Proportion Single Parent (N)
Fetal Death	6,805,928	6,847,730	6,914,753	6,914,730	6,915,207	6,915,375	6,915,463
Infant Death	6,600,191	6,640,759	6,705,755	6,705,731	6,706,197	6,706,365	6,706,450
Preterm Birth	6,467,638	6,507,161	6,570,137	6,570,114	6,570,565	6,570,731	6,570,808
Smoking	6,559,239	6,599,604	6,664,397	6,664,373	6,664,838	6,665,006	6,665,089
BMI	5,764,787	5,800,338	5,857,262	5,857,238	5,857,647	5,857,799	5,857,862
Birthweight	6,522,516	6,562,502	6,626,662	6,626,639	6,627,092	6,627,258	6,627,339

**Table:** 

	Coefficient	95 % CI	
Economic Connectivity	−0.21∗∗∗	−0.23	−0.19
Infant Sex (ref = Female)			
Male	0.03∗	0.00	0.05
Health Insurance (ref = Medi-Cal)			
Private	−1.08∗∗∗	−1.13	−1.03
Other	−0.19∗∗∗	−0.28	−0.09
WIC (ref = No WIC)	−1.04∗∗∗	−1.08	−0.99
Maternal Age	−0.14∗∗∗	−0.15	−0.12
Maternal Age X Maternal Age	0.003∗∗∗	0.00	0.003
Maternal Education (ref= < High School)			
High School Graduate	−0.03	−0.07	0.01
> High School	−0.20∗∗∗	−0.25	−0.16
Race/Ethnicity (Ref = Non-Hispanic White)			
Non-Hispanic Black	0.52∗∗∗	0.47	0.57
Non-Hispanic Asian	−0.20∗∗∗	−0.26	−0.14
Hispanic	−0.18∗∗∗	−0.22	−0.14
Other	−0.13∗∗	−0.23	−0.03

† Coefficients in this logistic regression estimate log-odds, with the null value being 0; exponentiation of coefficients would provide the odds ratio.

∗p < 0.05, ∗∗p < 0.01, ∗∗∗p < 0.001.

**Table:** 

	Coefficient	95 % CI	
Clustering	0.01	−0.00	0.03
Infant Sex (ref = Female)			
Male	0.03∗	0.00	0.05
Health Insurance (ref = Medi-Cal)			
Private	−1.13∗∗∗	−1.19	−1.08
Other	−0.22∗∗∗	−0.31	−0.12
WIC (ref = No WIC)	−1.04∗∗∗	−1.09	−1.00
Maternal Age	−0.14∗∗∗	−0.15	−0.12
Maternal Age X Maternal Age	0.003∗∗∗	0.002	0.003
Maternal Education (ref= <High School)			
High School Graduate	−0.04	−0.08	0.00
>High School	−0.24∗∗∗	−0.29	−0.19
Race/Ethnicity (Ref = Non-Hispanic White)			
Non-Hispanic Black	0.57∗∗∗	0.52	0.62
Non-Hispanic Asian	−0.21∗∗∗	−0.27	−0.15
Hispanic	−0.14∗∗∗	−0.18	−0.10
Other	−0.13∗	−0.23	−0.03

^†^ Coefficients in this logistic regression estimate log-odds, with the null value being 0; exponentiation of coefficients would provide the odds ratio.

∗p < 0.05, ∗∗p < 0.01, ∗∗∗p < 0.001.

**Table:** 

	Coefficient	95 % CI	
Gini Coefficient	−0.04∗∗∗	−0.06	−0.02
Infant Sex (ref = Female)			
Male	0.03	−0.00	0.05
Health Insurance (ref = Medi-Cal)			
Private	−1.14∗∗∗	−1.20	−1.08
Other	−0.24∗∗∗	−0.33	−0.14
WIC (ref = No WIC)	−1.04∗∗∗	−1.09	−1.00
Maternal Age	−0.14∗∗∗	−0.15	−0.12
Maternal Age X Maternal Age	0.003∗∗∗	0.002	0.003
Maternal Education (ref= <High School)			
High School Graduate	−0.04	−0.08	0.00
>High School	−0.24∗∗∗	−0.28	−0.19
Race/Ethnicity (Ref = Non-Hispanic White)			
Non-Hispanic Black	0.57∗∗∗	0.51	0.62
Non-Hispanic Asian	−0.21∗∗∗	−0.27	−0.15
Hispanic	−0.14∗∗∗	−0.18	−0.10
Other	−0.15∗∗	−0.25	−0.05

^†^ Coefficients in this logistic regression estimate log-odds, with the null value being 0; exponentiation of coefficients would provide the odds ratio.

∗p < 0.05, ∗∗p < 0.01, ∗∗∗p < 0.001.

**Table:** 

	Coefficient	95 % CI	
Household Median Income	−0.13∗∗∗	−0.15	−0.11
Infant Sex (ref = Female)			
Male	0.03	−0.00	0.05
Health Insurance (ref = Medi-Cal)			
Private	−1.11∗∗∗	−1.16	−1.05
Other	−0.22∗∗∗	−0.31	−0.12
WIC (ref = No WIC)	−1.04∗∗∗	−1.09	−1.00
Maternal Age	−0.14∗∗∗	−0.15	−0.12
Maternal Age X Maternal Age	0.003∗∗∗	0.002	0.003
Maternal Education (ref= <High School)			
High School Graduate	−0.04	−0.07	0.01
>High School	−0.22∗∗∗	−0.26	−0.18
Race/Ethnicity (Ref = Non-Hispanic White)			
Non-Hispanic Black	0.54∗∗∗	0.49	0.59
Non-Hispanic Asian	−0.20∗∗∗	−0.26	−0.14
Hispanic	−0.15∗∗∗	−0.19	−0.11
Other	−0.16∗∗	−0.26	−0.06

^†^ Coefficients in this logistic regression estimate log-odds, with the null value being 0; exponentiation of coefficients would provide the odds ratio.

∗p < 0.05, ∗∗p < 0.01, ∗∗∗p < 0.001.

**Table:** 

	Coefficient	95 % CI	
Share Below Federal Poverty Level	0.12∗∗∗	0.10	0.14
Infant Sex (ref = Female)			
Male	0.03	−0.00	0.05
Health Insurance (ref = Medi-Cal)			
Private	−1.12∗∗∗	−1.17	−1.06
Other	−0.22∗∗∗	−0.31	−0.12
WIC (ref = No WIC)	−1.04∗∗∗	−1.09	−1.00
Maternal Age	−0.14∗∗∗	−0.15	−0.12
Maternal Age X Maternal Age	0.003∗∗∗	0.002	0.003
Maternal Education (ref= <High School)			
High School Graduate	−0.03	−0.07	0.01
>High School	−0.22∗∗∗	−0.26	−0.17
Race/Ethnicity (Ref = Non-Hispanic White)			
Non-Hispanic Black	0.54∗∗∗	0.48	0.58
Non-Hispanic Asian	−0.21∗∗∗	−0.27	−0.15
Hispanic	−0.15∗∗∗	−0.19	−0.11
Other	−0.16∗∗	−0.26	−0.06

^†^ Coefficients in this logistic regression estimate log-odds, with the null value being 0; exponentiation of coefficients would provide the odds ratio.

∗p < 0.05, ∗∗p < 0.01, ∗∗∗p < 0.001.

**Table:** 

	Coefficient	95 % CI	
Share Non-Hispanic Black	0.05∗∗∗	0.04	0.07
Infant Sex (ref = Female)			
Male	0.03	−0.00	0.05
Health Insurance (ref = Medi-Cal)			
Private	−1.14∗∗∗	−1.20	−1.08
Other	−0.23∗∗∗	−0.33	−0.14
WIC (ref = No WIC)	−1.04∗∗∗	−1.09	−1.00
Maternal Age	−0.14∗∗∗	−0.15	−0.12
Maternal Age X Maternal Age	0.003∗∗∗	0.002	0.003
Maternal Education (ref= <High School)			
High School Graduate	−0.04	−0.08	0.00
>High School	−0.23∗∗∗	−0.28	−0.19
Race/Ethnicity (Ref = Non-Hispanic White)			
Non-Hispanic Black	0.53∗∗∗	0.48	0.58
Non-Hispanic Asian	−0.21∗∗∗	−0.27	−0.15
Hispanic	−0.14∗∗∗	−0.18	−0.10
Other	−0.15∗∗	−0.25	−0.05

^†^ Coefficients in this logistic regression estimate log-odds, with the null value being 0; exponentiation of coefficients would provide the odds ratio.

∗p < 0.05, ∗∗p < 0.01, ∗∗∗p < 0.001.

**Table:** 

	Coefficient	95 % CI	
Share Single Parent Household	0.11∗∗∗	0.09	0.13
Infant Sex (ref = Female)			
Male	0.03	−0.00	0.05
Health Insurance (ref = Medi-Cal)			
Private	−1.12∗∗∗	−1.17	−1.06
Other	−0.22∗∗∗	−0.31	−0.12
WIC (ref = No WIC)	−1.04∗∗∗	−1.09	−1.00
Maternal Age	−0.14∗∗∗	−0.15	−0.12
Maternal Age X Maternal Age	0.003∗∗∗	0.002	0.003
Maternal Education (ref= <High School)			
High School Graduate	−0.04	−0.08	0.00
>High School	−0.22∗∗∗	−0.27	−0.18
Race/Ethnicity (Ref = Non-Hispanic White)			
Non-Hispanic Black	0.52∗∗∗	0.47	0.57
Non-Hispanic Asian	−0.19∗∗∗	−0.25	−0.14
Hispanic	−0.14∗∗∗	−0.18	−0.10
Other	−0.16∗∗	−0.26	−0.06

^†^ Coefficients in this logistic regression estimate log-odds, with the null value being 0; exponentiation of coefficients would provide the odds ratio.

∗p < 0.05, ∗∗p < 0.01, ∗∗∗p < 0.001.

**Table:** 

	Main Model Coef. (SE)	Robust Model with trimmed EC and Clustering Z-scores Coef. (SE)	Robust Model including Outcome Outliers Coef. (SE)
Economic Connectivity			
Fetal Death	−0.21 (0.01)	−0.21 (0.01)	
Infant Death	−0.23 (0.01)	−0.23 (0.01)	
Preterm Birth	−0.12 (0.00)	−0.12 (0.00)	
Smoking	−0.12 (0.00)	−0.12 (0.00)	
BMI (kg/m^2^)	−0.87 (0.03)	−0.87 (0.03)	−0.94 (0.03)
Birthweight (grams)	7.56 (0.83)	7.45 (0.83)	6.7 (0.89)
**Clustering**			
Fetal Death	0.01 (0.01)	−0.01 (0.01)	
Infant Death	0.01 (0.01)	−0.02 (0.01)	
Preterm Birth	−0.00 (0.00)	−0.02 (0.01)	
Smoking	0.12 (0.00)	0.12 (0.00)	
BMI (kg/m^2^)	0.23 (0.02)	0.23 (0.03)	0.25 (0.02)
Birthweight (grams)	4.08 (0.79	9.19 (1.17)	4.64 (0.84)

**Table:** 

	Mean (Std Dev)	Minimum	Interquartile Range	Maximum	N
Economic Connectivity	0.86 (0.27)	0.23	0.66–1.04	1.71	8,333,366
Clustering	0.09 (0.01)	0.06	0.08–0.09	0.23	8,385,386
GINI Coefficient	0.42 (0.04)	0.00	0.39–0.45	0.74	8,466,914
Median HH Income ($)	61,388 (22,767)	2499	44,758–74,097	240,833	8,466,888
Proportion Poor	0.16 (0.09)	0	0.09–0.21	1.00	8,467,504
Proportion NH Black	0.06 (0.08)	0	0.02–0.08	0.85	8,467,709
Proportion Single Parent HH	0.30 (0.09)	0	0.24–0.36	1.00	8,467,821

## Data Availability

The authors do not have permission to share data.
